# The effects of a fat loss supplement on resting metabolic rate and hemodynamic variables in resistance trained males: a randomized, double-blind, placebo-controlled, cross-over trial

**DOI:** 10.1186/s12970-016-0125-z

**Published:** 2016-04-01

**Authors:** Bill I. Campbell, Ryan J. Colquhoun, Gina Zito, Nic Martinez, Kristina Kendall, Laura Buchanan, Matt Lehn, Mallory Johnson, Courtney St. Louis, Yasmin Smith, Brad Cloer

**Affiliations:** Exercise Science Program, Performance & Physique Enhancement Laboratory, College of Education, University of South Florida, Tampa, FL 33620 USA; Bodybuilding.com, Boise, ID 83713 USA

**Keywords:** Sports nutrition, Metabolism, Dietary supplement, Male physique enhancement, Weight loss fat loss

## Abstract

**Background:**

While it is known that dietary supplements containing a combination of thermogenic ingredients can increase resting metabolic rate (RMR), the magnitude can vary based on the active ingredient and/or combination of active ingredients. The purpose of this study was to examine the effects of a commercially available thermogenic fat loss supplement on RMR and hemodynamic variables in healthy, resistance trained males.

**Methods:**

Ten resistance-trained male participants (29 ± 9 years; 178 ± 4 cm; 85.7 ± 11 kg, and BMI = 26.8 ± 3.7) volunteered to participate in this randomized, double-blind, placebo controlled cross-over study. Participants underwent two testing sessions separated by at least 24 h. On their first visit, participants arrived to the laboratory after an overnight fast and a 24-h avoidance of exercise, and underwent a baseline RMR, HR, and BP assessment. Next, each participant ingested a thermogenic fat loss supplement (TFLS) or a placebo (PLA) and repeated the RMR, HR, and BP assessments at 60, 120, and 180 min post-ingestion. During the second visit the alternative supplement was ingested and the assessments were repeated in the exact same manner. Data were analyzed via a 2-factor [2x4] within-subjects repeated measures analysis of variance (ANOVA). Post-hoc tests were analyzed via paired samples t-tests. The criterion for significance was set at *p* ≤ 0.05.

**Results:**

A significant main effect for time relative to raw RMR data (*p* = 0.014) was observed. Post-hoc analysis revealed that the TFLS significantly increased RMR at 60-min, 120-min, and 180-min post ingestion (*p* < 0.05) as compared to baseline RMR values. No significant changes in RMR were observed for the PLA treatment (*p* > 0.05). Specifically, RMR was increased by 7.8 % (from 1,906 to 2,057 kcal), 6.9 % (from 1,906 to 2,037 kcal), and 9.1 % (from 1,906 to 2,081 kcal) in the TFLS, while the PLA treatment increased RMR by 3.3 % (from 1,919 to 1,981 kcal), 3.1 % (from 1,919 to 1,978 kcal), and 2.1 % (from 1,919 to 1,959 kcal) above baseline at 60, 120, and 180-min post ingestion, respectively. Additionally, the TFLS significantly elevated RMR at the 3-h time point as compared to the PLA treatment (2,081 vs 1,959 kcal, *p* = 0.034). A main effect for groups was observed for systolic blood pressure, and a significant interaction and main effect for time were observed for diastolic blood pressure. It should be noted that although changes in diastolic blood pressure were significant, all values stayed within normal clinical ranges (<80 mmHg).

**Conclusions:**

The TFLS led to significant elevations in RMR as compared to baseline. These elevations came with no adverse effect relative to resting heart rate, but a slight increase in blood pressure values. Taken on a daily basis, this TFLS may increase an individual’s overall energy expenditure, however; future studies should investigate if this leads to a reduction in fat mass loss over time.

## Background

One of the most popular categories of dietary supplements is weight loss supplements, or "fat burners". These supplements contain thermogenic ingredients aimed at increasing resting metabolic rate (RMR) and facilitating fat loss. Recent investigations have identified that commercially available thermogenic supplements and ingredients typically contained in such products can increase RMR in healthy subjects [1–3], and when taken chronically, may elicit favorable changes in body composition [4, 5].

Caffeine supplementation has previously been shown to enhance lipolysis and fat oxidation [6]. However, when combined with additional herbal ingredients, the combination appears to be more effective for increasing RMR [7]. The majority of thermogenic supplements contain a combination of dietary ingredients such as caffeine, green tea extract, and various herbal extracts that have been shown to increase metabolism [6–9], decrease body fat [4, 5], and increase markers of lipolysis [10, 11].

It is generally accepted that caffeine increases resting metabolic rate (RMR) through activation of β2 and β3 adrenergic receptors, as well as activation of cyclic AMP (cAMP) [7, 12], causing subsequent increases in circulating epinephrine and free fatty acids [13, 14]. Green tea extract (GTE), which contains high amounts of catechins polyphenols, is also found in many thermogenic supplements and has been shown to increase both energy expenditure and fat oxidation [7, 15–18]. Catechin polyphenols, like epigallocatechin gallate (EGCG), have been found to produce a sparing effect on noradrenaline, ultimately leading to increased levels of the catecholamine, which helps to stimulate cAMP [19]. These two ingredients together (caffeine and green tea extract) have been shown to significantly increase RMR beyond the individual capabilities of caffeine or GTE [7].

L-carnitine is another ingredient found in many weight loss products due to its role in fat metabolism. The primary function of L-carnitine it to transport long-chain fatty acids across the mitochondrial membrane [20]. Once inside, fatty acids can be degraded to acetyl-CoA through beta oxidation and proceed to the citric acid cycle. The idea behind L-carnitine supplementation for fat loss is that it can increase fat oxidation eventually leading to a gradual loss of body fat stores; however, there is limited supportive evidence for L-carnitine supplementation and weight loss [21].

One of the common concerns about using a thermogenic supplement is its effect on hemodynamic variables, such as heart rate (HR) and blood pressure (BP). Previous investigations have suggested that caffeine does have a stimulatory effect on systolic blood pressure (SBP), but the combination of additional ingredients in the product must be taken into consideration. Some trials have shown acute increases in HR and BP following ingestion of thermogenic supplements containing caffeine plus ephedra [22, 23]. Others have reported similar elevations in HR and BP following ingestion of a thermogenic product, even when ephedra was not present [1]. Of the ingredients found in the thermogenic product currently being investigated, there is some support suggesting caffeine and GTE can significantly increase energy expenditure without adversely affecting hemodynamic variables [8, 16, 24, 25].

While it is known that these ingredients can have a positive influence on RMR, the magnitude can vary based on the active ingredient and/or combination of active ingredients. Additionally, further safety evaluation is needed on multi-ingredient supplements that contain caffeine because of its potential effect on HR and BP. Therefore, the primary objective for this study was to determine the effects of the thermogenic fat loss supplement Iron Cuts™ on RMR in healthy, resistance-trained males. A secondary objective of this study was to determine the effects of this thermogenic fat loss supplement on resting HR and BP.

## Methods

### Participants

Ten healthy males (age: 29 ± 9 years; height: 178 ± 4 cm; bodyweight: 85 ± 11 kg; BMI: 26.8 ± 3.7) between the ages of 18 and 50 years volunteered to participate in this randomized, double-blind, placebo controlled cross-over study. All participants reported engaging in resistance exercise an average of four days per week. The research protocol was approved by the University of South Florida Institutional Review Board. Following an explanation of all risks and benefits associated with the experimental protocol, each participant gave his informed consent to participate in this study. Participants were screened for participation based on established criteria set forth by the American College of Sports Medicine [26]. In order to participate in the study, participants needed to be free from cardiovascular, pulmonary, and metabolic disease. Participants that were categorized as ‘high risk’ for cardiovascular disease according to the American College of Sports Medicine’s risk stratification were excluded from participation in the study. Participants were also excluded as a result of any intolerance or known allergy to the supplement ingredients.

### Experimental design

The study utilized a randomized, double blind, placebo controlled crossover design. Participants reported to the Performance and Physique Enhancement Laboratory following an overnight fast (a minimum of an 8-h fast) and a 24-h avoidance of exercise on two occasions separated by at least 24 h. The laboratory was climate controlled throughout the duration of the study with the following recorded variables: temperature = 21.3 °C; humidity = 50.4 %; and barometric pressure = 762 mmHg. After arriving to the laboratory, a coin was flipped to randomly determine the order of the dietary supplement ingestion. If the participant were randomized to ingest the thermogenic fat loss supplement (Iron Cuts™) on the first testing session, they would ingest the alternate treatment (placebo) on the second and final testing session. Likewise, if the participant were randomized to ingest the placebo treatment on the first testing session, they would ingest the alternate treatment (Iron Cuts™) on the subsequent laboratory visit. Testing sessions for both laboratory visits occurred between the hours of 6:30 am and 8:00 am, with the majority of all assessments beginning at 7 am.

### Testing sessions

Upon arriving to the laboratory, participants were encouraged to visit the restroom to void their bladders of urine. Next, body weight was measured on a physician beam scale (Health-O-Meter™, Model 402KL, McCook, IL, USA) and then the participant sat in a reclined position with their feet elevated for a 5-min period. After sitting quietly for 5 min, participants had their resting heart rate and blood pressure recorded using an automated, oscillometric blood pressure monitor (Omron 5 series Model BP742, Lake Forest, IL, USA). This method of automated, oscillometric blood pressure measurement has been validated in the scientific literature [27]. Heart rate and blood pressure were measured in triplicate and the average of the three readings was recorded.

Next, the participant remained in a reclined position for an additional 5 min prior to the resting metabolic rate (RMR) measures. All RMR measures were made using a Cosmed FitMate Pro™ (Cosmed, Italy). The FitMate Pro™ contains a turbine flow meter for measuring ventilation and a galvanic fuel cell oxygen sensor for analyzing the fraction of oxygen in the expired gases. To sample the expired air, a facemask was placed over the participant’s face and was attached to the turbine flow meter. The device uses standard metabolic formulas to calculate oxygen uptake, and energy expenditure is calculated using a fixed respiratory quotient (RQ) of 0.85. The FitMate Pro™ also conducts a self-calibration before each measurement, and at two other times (5 and 10 min) during the 15-min RMR assessment. The device has been validated with a Douglas bag for non-obese and obese subjects and was found to calculate RMR accurately (*r* = 0.97, *p* = 0.579) [28]. Intra and inter-day test-retest correlation calculated for the device used in the present study were as follows: intra-day RMR Pearson correlation was *r* = 0.96 (*p* < 0.01) and the inter-day RMR Pearson correlation was *r* = 0.90 (*p* < 0.01). Intra-day RMR ICC was 0.981 and the inter-day RMR ICC was 0.946.

At baseline, two consecutive RMR tests were conducted and the lower of the two measured RMR values was recorded as the baseline RMR value. During the RMR test, the participant was instructed to relax during the test, to breathe normally, and to remain as still as possible for the duration of the 15-min test. The first 5 min of data collection was discarded [29] and the final 10 min of data collected was used in the calculation of the resting metabolic rate.

**Fig. 1:**
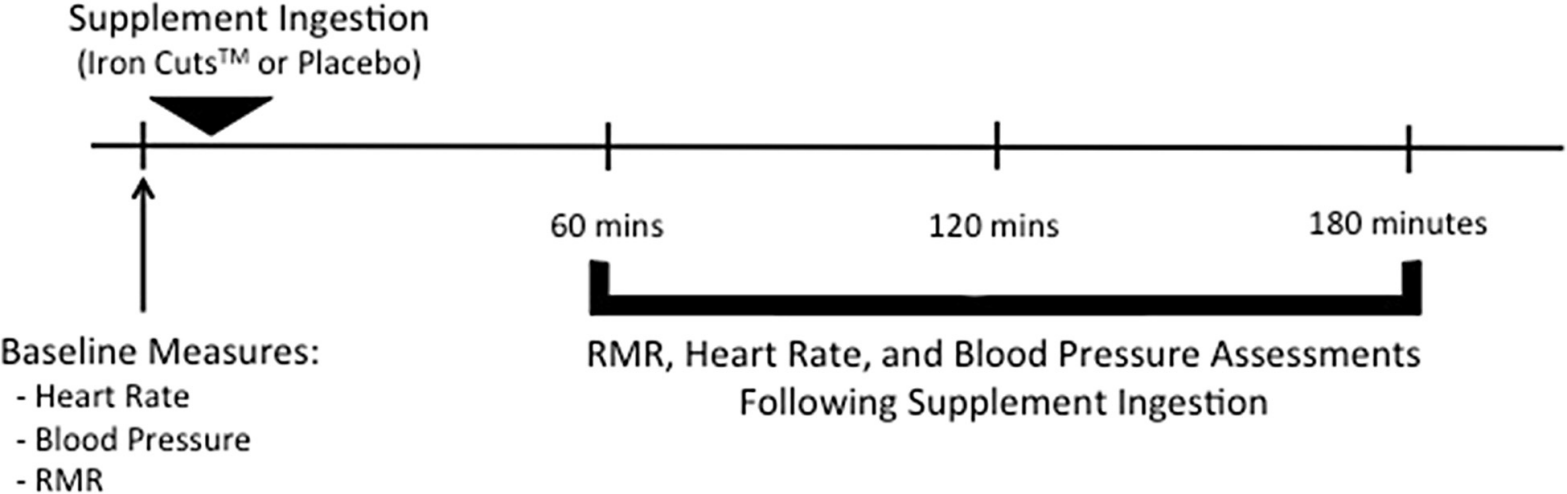
Overview of testing sessions

After baseline RMR was established, the participant ingested three capsules of either the thermogenic fat loss supplement (Iron Cuts™) or placebo treatment. After ingestion of the supplement treatments, three more heart rate, blood pressure, and RMR assessments were conducted at 1-h, 2-h, and 3-h post ingestion. Figure 1 presents an overview of the study test sessions.

### Supplement

The thermogenic fat loss supplement (Iron Cuts™) treatment and placebo were ingested in capsule form, and three capsules were ingested per dose. Capsules were identical in appearance and taste. The ingredients in the thermogenic fat loss supplement treatment (Iron Cuts™) are presented in Table 1, while the placebo contained only inert ingredients (650 mg of maltodextrin and 88.8 mg of hemp protein). Following the completion of two baseline RMR tests, the participant ingested three capsules of the thermogenic fat loss supplement or the placebo treatment with eight ounces of water. Supplement ingestion was in the presence of research personnel for all testing sessions.

**Table 1:** Thermogenic Fat Loss Supplement Ingredients

Supplement Facts		
Serving Size: 3 Capsules		
Servings Per Container: 40		
	Amount Per Serving	% DV^a^
Vitamin D (as Cholecalciferol)	400 IU	100 %
Chromium (as Chromium Picolinate)	50 mcg	42 %
Thermogenic & Fat Metabolizer	930 mg	**
L-Carnitine Tartrate, Green Tea (Camellia Sinensis) Leaf Extract, Caffeine Anhydrous, Panax Ginseng Root Powder, N-Acetyl-L-Tyrosine, Thermodiamine™ (98 % Evodiamine), Vinpocetine, Inositol.		
Muscle Building Maximizer	900 mg	**
Maca 4:1 (Lepidium meyenii) Root Extract, AminoShield® Eriobotrya japonica Leaf Extract Proprietary Blend of 20 % Pentacyclic Triterpenoids, Alpha Lipoic Acid, Boron Citrate, Fenugreek (Trigonella Foenum Graecum) Seed Extract 50 % Saponins, Pumpkin Seed (Cucurbita Moschata Poiret) Extract.		
Estrogen & Cortisol Metabolizer	383 mg	**
Gymnema Sylvestre Leaf Extract, Grape (Vitis vinifera) Seed Extract, Diindolylmethane (DIM), Cinnamon (Cinnamomum cassia) Bark powder, Banaba (Lagerstroemia speciosa) Leaf Extract 1 % Corosolic Acid, Chromium Picolinate.		

^a^Percent Daily Value Based on a 2,000 Calorie Diet

**Daily Value Not Established

### Statistical analysis

Statistical analyses of the data were analyzed via a 2-factor treatment by time [2x4] within-subjects repeated measures analysis of variance (ANOVA) using SPSS version 22.0. If sphericity could not be assumed, a Huynd-Feldt correction was used to produce a more critical *F*-value. Post-hoc tests (comparisons between treatments at each time point [baseline and 60-min, 120-min and 180-min post ingestion and comparisons of post-supplement ingestion with baseline measures within each treatment) were analyzed via paired samples t-tests. Incremental area under the curve (AUC) was calculated for each treatment (thermogenic supplement and placebo) using the trapezoidal method as described by Brouns et al. [30]. AUC was determined by measuring the increase in RMR above baseline over the three-hour assessment period. A paired samples t-test was used to determine AUC differences between the two treatments. A criterion α-level of *p* ≤ 0.05 was used to determine statistical significance.

## Results

### Resting metabolic rate

For both supplement treatments, a Shapiro-Wilk’s test (*p* > 0.05) [31] and a visual inspection of their histograms, normal Q-Q plots and box plots showed that the resting metabolic rate scores were normally distributed. Skewness scores [displayed as statistic (standard error)] ranged from 0.013 to 0.36 (0.69) and kurtosis scores [displayed as statistic (standard error)] ranged from –1.52 to 1.14 (1.33) for the Iron Cuts™ treatment; skewness scores ranged from 0.58 to 0.96 (0.69) and kurtosis scores ranged from -0.28 to 2.06 (1.33) for the placebo treatment [32]. The standardized (z scores) skewness and kurtosis coefficients for both treatments were within the range of ±1.5 [33].

**Fig. 2:**
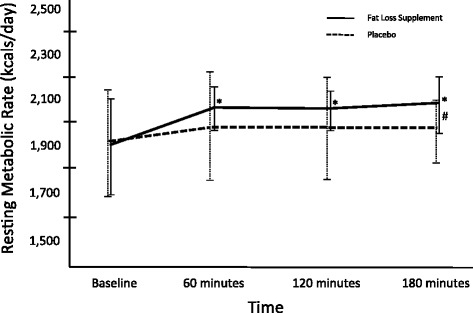
Change in resting metabolic rate (RMR) from baseline to 3-h post ingestion. Data is expressed as mean (SD). * = significant increase in RMR as compared to baseline for thermogenic fat loss supplement treatment (p < 0.05). # = significant difference between thermogenic fat loss supplement and placebo three hours post-ingestion (p < 0.05)

**Fig. 3:**
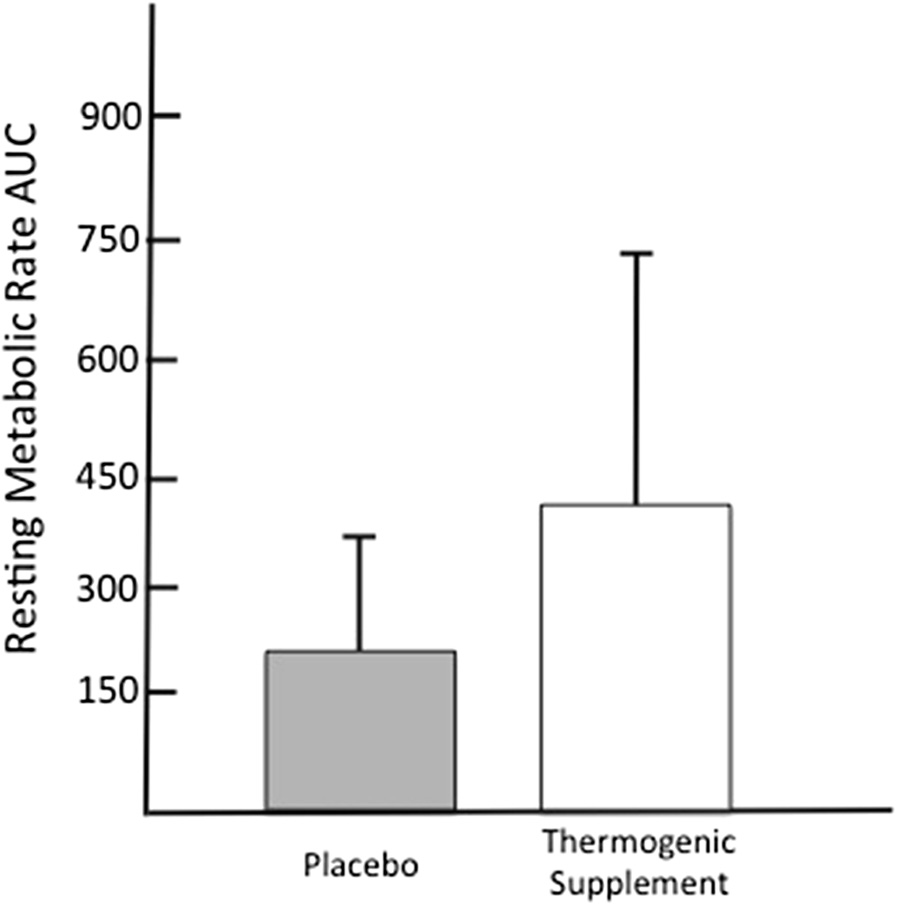
Baseline Subtracted Area Under the Curve (kcals/3 h)

Paired samples t-test revealed no significant difference in baseline RMR between the two treatments. Repeated measures ANOVA revealed a significant main effect for time (*p* = 0.014), but no treatment x time (*p* = 0.224) or main effect for group (*p* = 0.145). Post-hoc analysis revealed that the thermogenic fat loss supplement treatment (Iron Cuts™) significantly increased RMR at 60 min, 120 min, and 180 min post ingestion (*p* = 0.007, 0.046, 0.010, respectively), as compared to baseline RMR values (Fig. 2). No significant changes in RMR were observed for the placebo treatment in comparison with baseline values. Specifically, RMR was increased by 7.8 %, 6.9 %, and 9.1 % in the thermogenic fat loss supplement, while the placebo treatment increased RMR by 3.3 %, 3.1 %, and 2.1 % above baseline at 60, 120, and 180-min post ingestion, respectively. Also, when comparing the groups at each time point, the thermogenic fat loss supplement significantly elevated RMR at the 3-h time point as compared to the placebo treatment (thermogenic fat loss supplement RMR = 2,081 ± 114 kcals/day; placebo treatment RMR = 1,959 ± 148 kcals/day; *p* = 0.034), elevating RMR 6.2 % above the placebo treatment at this point in time. Relative to AUC comparisons, no significant differences (*p* = 0.131) were observed between the thermogenic fat loss supplement and placebo treatments (Fig. 3).

### Hemodynamic response

There were no group x time interaction effects (*p* = 0.130) and no main effects for group (*p* = 0.135) or time (*p* = 0.110) relative to heart rate for the two supplement treatments. For systolic blood pressure (SBP), no group x time interaction effect (*p* = 0.433) or main effect for time (*p* = 0.136) was observed, but a main effect for group (*p* = 0.024) was revealed. For diastolic blood pressure (DBP), a significant group x time interaction (*p* = 0.013) and a main effect for time (*p* = 0.005) were observed, but no main effect for group (*p* = 0.068). Raw data for RMR and hemodynamic variables is summarized in Table 2.

**Table 2:** Resting metabolic rate and hemodynamic summary data

	Baseline		60 minutes		120 minutes		180 minutes	
	TFLS	Plac	TFLS	Plac	TFLS	Plac	TFLS	Plac
Resting Metabolic Rate (kcals/day)	1,906 (195)	1,919 (219)	2,057* (110)	1,981 (235)	2,037* (87)	1,978 (226)	2,081*,** (114)	1,959** (148)
Heart Rate (beats/min)	57.5 (9)	55.1 (9)	54.8 (10)	52.2 (10)	55.6 (6)	52.7 (9)	58.3 (8)	51.8 (12)
Systolic Blood Pressure (mmHg)	122 (11)	121 (12)	126 (11)	120 (12)	128 (12)*	123 (15)	127 (11)*	121 (12)
Diastolic Blood Pressure (mmHg)	66 (8)	69 (9)	76 (12)*^,^**	70 (9)**	75 (10)*^,^**	70 (8)**	73 (10)*	73 (8)

*TFLS* fat loss supplement. *Plac* placebo. Data is presented as mean (± standard deviation). * = *p* < 0.05 within group change as compared to baseline value. ** = *p* < 0.05 between groups difference at same time point

## Discussion

The aim of this study was to examine the effects of a multi-ingredient thermogenic fat loss supplement on RMR and hemodynamic function in resistance-trained males. Findings from the study show that acute ingestion of the thermogenic fat loss supplement, Iron Cuts™, leads to a significant increase in RMR when compared to a placebo in healthy men. The observed increase in RMR occurred with a slight increase in SBP and DBP, while HR was not affected by the thermogenic supplement.

Previous research has supported the idea that ingestion of thermogenic supplements containing caffeine in combination with various additional ingredients can acutely increasing RMR. In the current study, the thermogenic fat loss supplement treatment experienced a greater elevation in RMR values compared to baseline, whereas the placebo treatment did not. Caffeine, in combination with other herbal ingredients, has been shown to increase RMR for up to three hours post ingestion [1, 2, 9, 25]. In agreement with these findings, the current study demonstrated a significant 7.8 %, 6.9 %, and 9.1 % increase in RMR above baseline 60, 120, and 180 min post ingestion, respectively. The placebo treatment, however, experienced only a 3.3 %, 3.1 %, and 2.1 % increase in RMR 60, 120, and 180 min post ingestion, respectively.

Caffeine is a popular ingredient used in many thermogenic supplements due to its ability to increase energy expenditure. Caffeine alone [8, 34], caffeine plus green tea extract (GTE) [7, 34, 35], and caffeine used in combination with other herbal ingredients [2] have been shown to significantly elevate RMR (when compared to placebo). Taylor et al. [9] reported a 14.4 % increase in RMR following ingestion of a coffee beverage containing additional caffeine, GTE, niacin, and garcinia cambogia. Furthermore, Wilborn et al. [25] reported a 15.5 % increase in RMR three hours post-ingestion of a thermogenic product containing caffeine, GTE, and yohimbine-HCL. In agreement with these findings, Dalbo et al. [36] reported a 10.5 % increase in RMR over a three hour period following ingestion of a thermogenic drink containing caffeine and GTE. While the increase in RMR observed in the current study was lower than that of previous studies, differences could be attributed to the dosages used, the combinations of ingredients, and the concentrations of individual ingredients. The dose of caffeine in the current product (200 mg) was lower than that of the Taylor et al. and Wilborn et al. studies (300–400 mg), providing possible explanation as to the differences observed for RMR elevations.

Green tea extract, whether used on its own or in combination with caffeine, has been shown to increase RMR. However, the combination of GTE and caffeine can significantly increase catecholamine release (which stimulates glycogen and triglyceride catabolism), leading to further increases in RMR [1]. Therefore, the observed increase in RMR in the current study is likely the result of the combination of the two stimulants. It is unlikely that L-carnitine played a role in increasing energy expenditure. While it may be possible that L-carnitine has an effect on fat metabolism after several months of ingestion, it is too early to draw any conclusions based on the acute dose used in the current study.

The secondary purpose of this study was to examine the effects of Iron Cuts™ on resting hemodynamic variables. Acute ingestion of Iron Cuts™ did not significantly alter resting heart rate. However, there was a slight, yet significant, increase in both systolic and diastolic blood pressure. It has been demonstrated that long-term consumption of caffeine has minimal effect on hemodynamic function [8, 37, 38]; however, there are some studies showing acute increases in blood pressure following ingestion of a thermogenic supplement [1, 22]. Similar to previous studies, the present study observed a significant increase in blood pressure across time, with the dietary supplement treatment causing an increase in systolic blood pressure at both the 120 and 180-min time points and an elevation in diastolic blood pressure values over the three-hour testing period. While ingestion of the thermogenic fat loss supplement caused elevations in diastolic blood pressure at the one and two hour postingestion time points, it is important to note that the diastolic blood pressure values remained within normal clinical ranges throughout the three-hour intervention period.

## Conclusion

The major findings from this study indicate that ingestion of a thermogenic fat loss supplement (Iron Cuts™) containing approximately 200 mg of caffeine, green tea extract, and other ingredients can significantly increase RMR over a three hour time period in healthy males. These elevations came with no adverse effects relative to resting heart rate and only slight increases in blood pressure values. Although the thermogenic fat loss supplement resulted in an elevation in RMR, at this time we are not able to conclude whether this can lead to actual fat loss over time in this population. Future studies should investigate the effectiveness and safety of ingesting the dietary supplement over a longer period of time (several weeks) to determine if reductions in fat mass are observed.
